# Effect of phenylephrine vs. ephedrine on frontal lobe oxygenation during caesarean section with spinal anesthesia: an open label randomized controlled trial

**DOI:** 10.3389/fphys.2014.00081

**Published:** 2014-03-03

**Authors:** Visti T. Foss, Robin Christensen, Kim Z. Rokamp, Peter Nissen, Niels H. Secher, Henning B. Nielsen

**Affiliations:** ^1^Department of Anaesthesia, Næstved HospitalNæstved, Denmark; ^2^Musculoskeletal Statistics Unit, Department of Rheumatology, The Parker Institute, Frederiksberg Hospital, University of CopenhagenCopenhagen, Denmark; ^3^Department of Anaesthesia, Rigshospitalet, University of CopenhagenCopenhagen, Denmark

**Keywords:** cerebral autoregulation, drug effect, heart rate, fetal, near infrared spectroscopy, vasoconstrictor agents

## Abstract

**Background:** During caesarean section spinal anesthesia may provoke maternal hypotension that we prevent by administration of phenylephrine and/or ephedrine. Phenylephrine is however reported to reduce the near infrared spectroscopy-determined frontal lobe oxygenation (ScO_2_) but whether that is the case for patients exposed to spinal anesthesia is not known.

**Objectives:** To evaluate the impact of phenylephrine vs. ephedrine on ScO_2_during caesarean section with spinal anesthesia in a single center, open-label parallel-group study with balanced randomization of 24 women (1:1). Secondary aims were to compare the effect of the two drugs on maternal hemodynamics and fetal heart rate.

**Intervention:** Ephedrine (0.8–3.3 mg/min) *vs.* phenylephrine infusion (0.02–0.07 mg/min).

**Results:** For the duration of surgery, administration of ephedrine maintained ScO_2_ (compared to baseline +2.1 ± 2.8%; mean ± SE, while phenylephrine reduced ScO_2_ (−8.6 ± 2.8%; *p* = 0.005) with a 10.7% difference in ScO_2_between groups (*p* = 0.0106). Also maternal heart rate was maintained with ephedrine (+3 ± 3 bpm) but decreased with phenylephrine (−11 ± 3 bpm); difference 14 bpm (*p* = 0.0053), but no significant difference in mean arterial pressure (*p* = 0.1904) or CO (*p* = 0.0683) was observed between groups. The two drugs also elicited an equal increase in fetal heart rate (by 19 ± 3 vs. 18 ± 3 bpm; *p* = 0.744).

**Conclusion:** In the choice between phenylephrine and ephedrine for maintenance of blood pressure during caesarean section with spinal anesthesia, ephedrine maintains frontal lobe oxygenation and maternal heart rate with a similar increase in fetal heart rate as elicited by phenylephrine.

**Trial registration:** Clinical trials NCT 01509521 and EudraCT 2001 006103 35.

## Introduction

Spinal anesthesia is used for caesarean section although it is commonly associated with hypotension. In addition to tilting patient to the left and providing i.v. fluids, we use ephedrine and/or phenylephrine to prevent or correct hypotension that could result in, e.g., dizziness, nausea, and vomiting and also hinder adequate perfusion of the child (Berlac and Rasmussen, 2005; Cyna et al., 2006; Saravanan et al., 2006; Ngan Kee et al., 2008a).

The effect of ephedrine and phenylephrine on maternal and fetal hemodynamics has been investigated extensively (Mercier et al., 2001; Saravanan et al., 2006; Langesaeter et al., 2008; Ngan Kee et al., 2008a) There seems to be no difference between the two drugs in regards to preventing hypotension following spinal anesthesia for caesarean section (Cyna et al., 2006) However, fetal tachycardia appears to be more frequent with the use of ephedrine, or combined ephedrine and phenylephrine than with the use of phenylephrine (Wang et al., 2011) Furthermore, the effect of phenylephrine on fetal acid base status seems to be more favorable than manifested with the use of ephedrine (Vesser et al., 2012).

On the other hand, near infrared spectroscopy (NIRS) determined cerebral (frontal lobe) oxygenation (ScO_2_) decreases when hypotension is supported by phenylephrine (Nissen et al., 2009a, 2010; Ogoh et al., 2011; Meng et al., 2011a) while it is maintained with the use of ephedrine (Nissen et al., 2010; Meng et al., 2011a; Ogoh et al., 2011) We investigated how phenylephrine and ephedrine influence ScO_2_ when used to prevent maternal hypotension during caesarean section performed under spinal anesthesia where phenylephrine may hinder accumulation of blood in the blocked area of the body and thereby support cardiac output (CO)(Cannesson et al., 2012) and in turn ScO_2_. Thus, we hypothesized that administration of both ephedrine and phenylephrine would maintain ScO_2_ in patients undergoing caesarean section with spinal anesthesia since an increase in vascular tone can enhance venous return and thereby support cardiac preload and CO during hypovolemia as demonstrated in anesthetized pigs (Cannesson et al., 2012) In this randomized clinical trial the primary outcome was to evaluate the ScO_2_ response to administration of ephedrine vs. phenylephrine. Secondary aims were to evaluate changes in maternal hemodynamics and in fetal heart rate. Umbilical cord blood gas variables were also determined.

## Methods

For this single center, open label, parallel-group study with balanced randomization (1:1), written informed consent was obtained from healthy women undergoing elective caesarean section during spinal anesthesia after approval by the local ethics committee (SJ—271) and the Danish medicine agency (NCT 01509521; EudraCT 2001 006103 35). The Good Clinical Practice (GCP) unit at the University of Copenhagen monitored the trial conducted from April 2012 to July 2012 at Næstved Hospital. Eligible participants were older than 18 years in ASA group I or II, 160–180 cm and with a single pregnancy. Patients with either preeclampsia, non-singleton pregnancy, HELLP-syndrome (hemolysis, elevated liver enzyme, low platelet count), elevated serum bilirubin, or reported allergy to ephedrine or phenylephrine were excluded from the study.

## Preparation

Upon arrival to the operating theater, the patient was provided with 500 mL isotonic saline, tilted 15° to the left and nasal supplementation of oxygen (2 L/min) was established. A cuff was applied to the third finger of the left hand and heart rate (HR), systolic (SBP), diastolic (DBP) and mean arterial (MAP) blood pressures were determined by Nexfin (BMEYE, The Netherlands, Amsterdam). Thus stroke volume (SV) was determined from the pressure curve using Modelflow (Bogert and van Lieshout, 2005) that takes sex, age, and weight into account. CO (SV times HR) and total peripheral resistance (TPR; MAP divided by CO) were calculated. Nexfin data were obtained on a beat to beat basis and averaged over 15 s every 2.5 min. Fetal HR was obtained by Doppler (Sonicaid Dopplers, Luton, Huntleigh, UK) and averaged over 15 s from before spinal anesthesia (baseline) and after spinal anesthesia (2.5–5 min), during surgery (7.5–17.5 min), and after delivery (13–28 min).

## Spinal anesthesia

With 2.4 mL bupivacaine 0.5% (12 mg) and 10 μg fentanyl spinal anesthesia was established using a 27 or 25 G pencil point needle at the L2–L3 or L3–L4 intervertebral space. Spinal anesthesia was administered in the right lateral position in 21 patients and in three patients spinal anesthesia was provided in a seated position. Surgery started when the sensory block included the T5 dermatome as indicated by the loss of sensation to application of cold to the skin.

## Frontal lobe oxygenation

ScO_2_ was monitored by NIRS (INVOS 3100 Cerebral Oxymeter, Somanetics, Troy, USA). Optodes were placed on both sides of the forehead immediately below the hairline and secured with a headband that also served to seal ambient light. The NIRS-determined mean ScO_2_ is based on optodes that emit and detect near-infrared light at two wavelengths (730 and 810 nm) and ScO_2_ is calculated as the ratio between oxyhemoglobin and total hemoglobin. The signal detector closest to the light source (3 cm) is considered the “shallow detector” and used to attenuate influence from superficial tissue, while the detector 4 cm from the light source is considered to detect light from “deep tissue.” The distance between the source and detectors is considered sufficient for light to reach the brain (Choi et al., 2004) Thus it was assumed that values are accounted for predominantly by hemoglobin in the frontal lobe cortex, although a contribution from the skin is acknowledged (Davie and Grocott, 2012).

## Interventions and randomization

The patients were allocated randomly into two groups (ephedrine or phenylephrine) of 12 using sequentially numbered, opaque, sealed envelopes prepared by an individual not involved in the study. Dose equivalence between phenylephrine (0.1 mg/mL) and ephedrine (5 mg/mL) was chosen in according to previous trials and recommendations (Saravanan et al., 2006; Ngan Kee et al., 2008b; Das et al., 2011).

After spinal anesthesia, the infusion was started (20 ml/h; 1.6 mg/min for ephedrine and 0.03 mg/min for phenylephrine) and adjusted to maintain SBP. If SBP increased from baseline by 10–20%, the infusion was reduced to 10 mL/h and paused if SBP increased more than 20%. Conversely, if SBP decreased by 10–20%, the infusion was increased to 40 mL/h. A bolus of either 10 mg ephedrine or 0.2 mg phenylephrine was to be administered if SBP decreased by more than 20%, or if the patient complained of symptoms of hypotension (dizziness, vomiting, faintness, nausea). At delivery the infusion was reduced to 10 mL/h and terminated 5–15 min thereafter. Data collected included Apgar score, and umbilical arterial and venous blood gas variables from a double-clamped cord segment. Further, data pertaining to the duration of surgery, volume of saline administered and the vasopressor dosages used are mentioned.

## Outcome measures

The primary outcome is expressed as the percentage ScO_2_ change from baseline, with baseline defined as rest prior to spinal anesthesia (time 0 min). The secondary outcomes variables (maternal hemodynamics and fetal heart rate) were also expressed as the change from baseline and all observations were continued until the end of surgery.

## Statistical analysis

Power calculations based on former studies (Nissen et al., 2010; Kim et al., 2000) revealed that in order to detect a statistically significant difference between means of 57 and 67% in ScO_2_, assuming a common standard deviation of 7%, a sample size of *n* = 9 per group was required to obtain a power of a least 0.8 (β = 20%; two-tailed α = 5%). To compensate for potential dropouts and missing data (25%) the sample size was increased to 12 participants per group. Data analyses were according to a pre-established plan using SAS software (v. 9.2; SAS Institute Inc., Cary, NC, USA). Descriptive statistics and tests are reported in accordance with the “Enhancing the QUAlity and Transparency Of health Research” (EQUATOR) network: the CONSORT Statement (Schulz et al., 2010). In order to evaluate data distribution of the outcome and statistical models, inspection was used to suggest whether the assumption of normality was reasonable. The PROC UNIVARIATE statement was used to summarize descriptive data. If the assumption of normality was not reasonable, we analyzed the data with the nonparametric Wilcoxon Rank Sum test using PROC NPAR1WAY; and the median difference was reported. The 95% confidence limit was estimated from an approximated standard error, based on the Wilcoxon *p*-value from a Wald-*Z*-test.

To analyse the longitudinal element of the study objectives, a linear approach was used for repeated measurements, using the procedure PROC MIXED based on restricted maximum likelihood (REML) estimates of the variables (Littell et al., 2000). The factor *Subject* was applied as a random effect factor. Assessment of the treatment and time effects tested possible interaction and both treatment and time were included as systematic factors using the baseline value as co-variate to reduce random variation and increase power. Unless stated otherwise, results are expressed as the difference between the group means and 95% CI with the associated *p*-values, based on the mixed linear model. The average change from the baseline during the study in each group was analyzed based on the mixed model with only the main effects of group and time without taking interaction into account. All comparisons were two tailed and *p* < 0.05 was considered statistically significant.

## Results

### Patients

**Figure 1:**
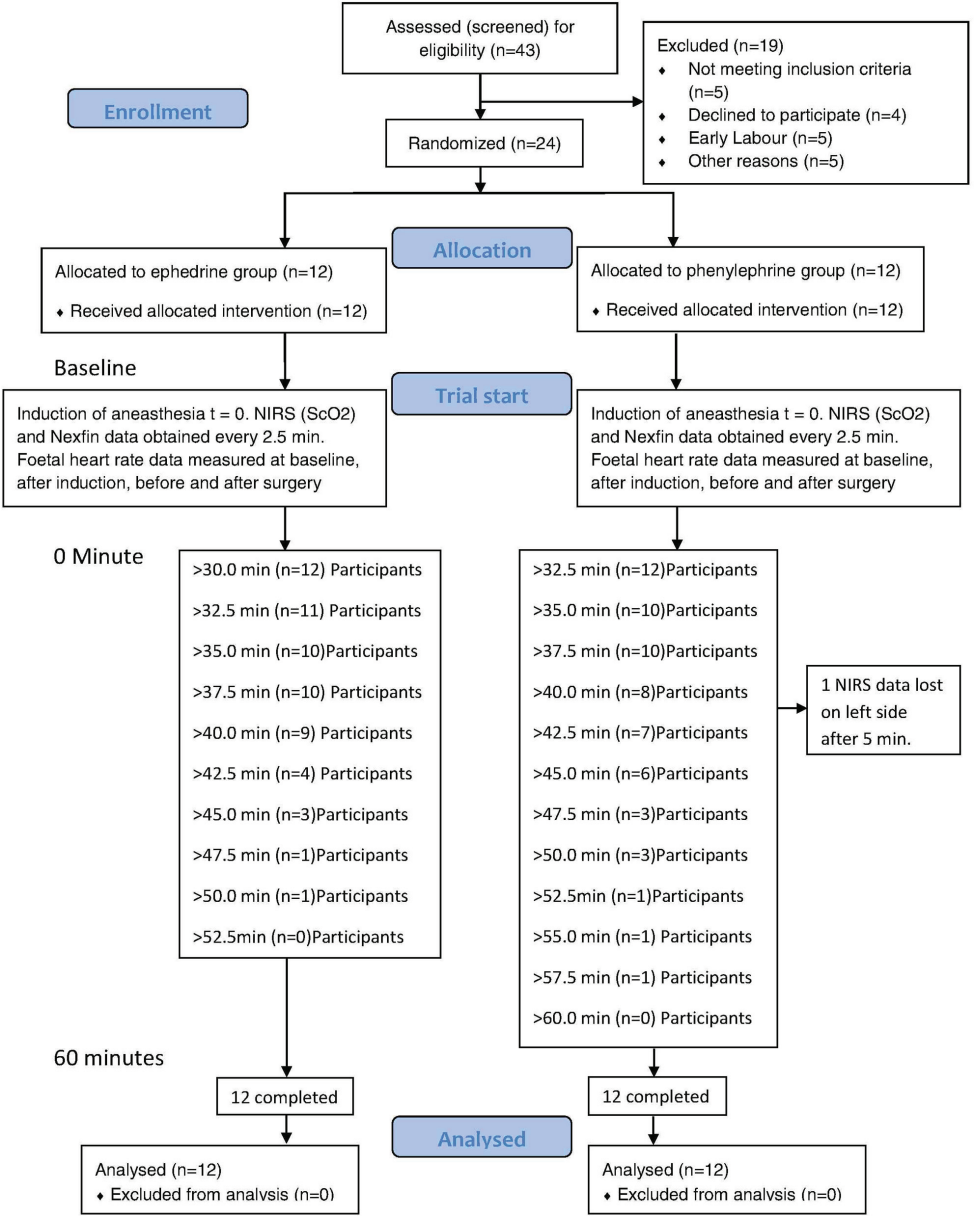
**Trial profile**.

Written informed consent to participate in the study was obtained from 33 patients; 24 of whom were subsequently randomized (Figure 1) and the randomized patients had a body mass index of 30.1 (5.4) mean (SD), range 21.6–41.6 kg/m^2^ (Table 1).

**Table 1:** **Patient characteristics, cardiovascular values, fetal heart rate, and frontal lobe oxygenation at baseline**.

	**Ephedrine ***N*** = **12****	**Phenylephrine ***N*** = **12****	**Total ***N*** = **24****
Age (years)	30.8 ± 5.5	33.0 ± 3.7	31.9 ± 4.7 (21–39)
Height (cm)	166 ± 6	168 ± 7	167 ± 6 (160–180)
Weight (kg)	89 ± 20	81 ± 8	85 ± 15.5 (67–125)
Body mass index (kg/m2)	32.2 ± 6.3	28.1 ± 3.2	30.1 ± 5.4 (21.6–41.6)
Systolic pressure (mmHg)	135 ± 20	137 ± 14	136 ± 17 (104–168)
Diastolic pressure (mmHg)	83 ± 9	80 ± 13	81 ± 11 (62–105)
Mean arterial pressure (mmHg)	102 ± 11	101 ± 12	101 ± 11 (82–122)
Heart rate (bpm)	95 ± 18	89 ± 21	92 ± 19 (59–132)
Stroke volume (ml)	97 ± 21	105 ± 20	101 ± 20 (59–149)
Cardiac output (L/min)	8.9 ± 1.1	9.0 ± 2.1	8.9 ± 1.6 (5.7–13.2)
Total peripheral resistance (dyn s cm^−5^)	933 ± 162	946 ± 262	940 ± 213 (570–1530)
Fetal heart rate (bpm)	138 ± 9	137 ± 9	138 ± 9 (120–150)
Frontal lobe oxygenation (%)	67 ± 10	64 ± 7	66 ± 8 (56–91)

Values are means ± SD. (range: minimum and maximum).

### Frontal lobe oxygenation

**Figure 2:**
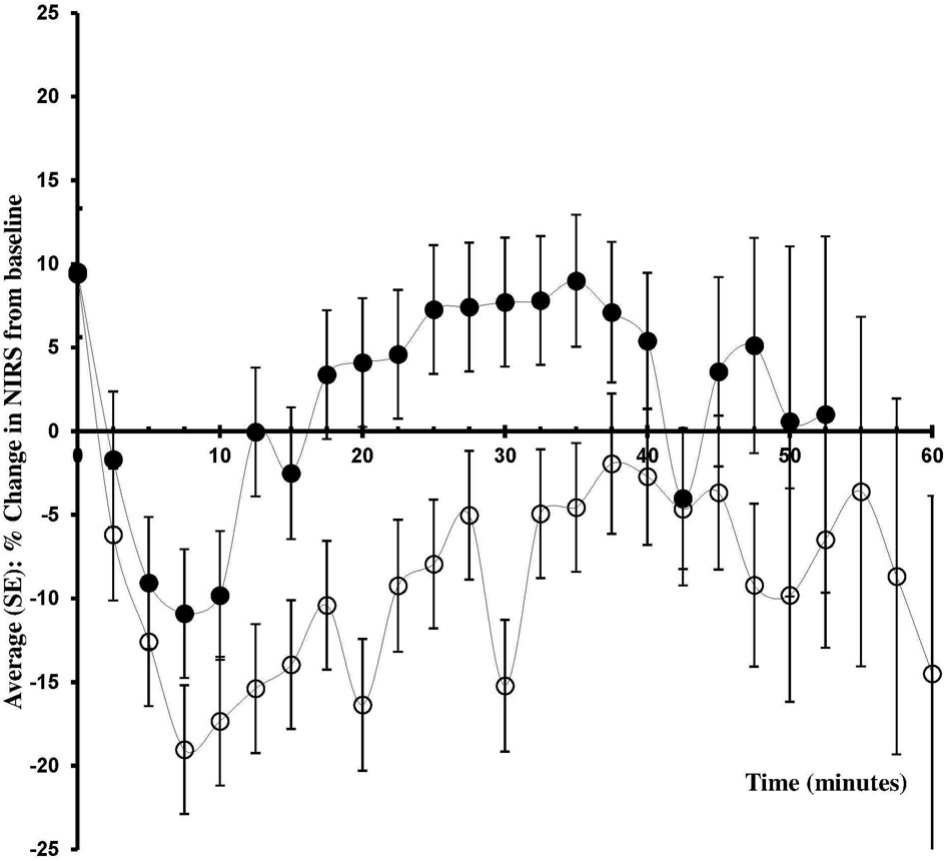
**Change in ScO_2_ from baseline**. Changes in frontal lobe oxygenation (ScO2; % from baseline) during caesarean section with spinal anesthesia. Patients received either phenylephrine (*n* = 12; open circles) or ephedrine (*n* = 12; black circles).

In patients allocated to administration of phenylephrine ScO_2_ decreased by −8.6 ± 2.8% while in patients randomized to infusion of ephedrine, ScO_2_ was not affected (+2.1 ± 2.8%). Comparing the values determined in the two groups, the difference in ScO_2_ was 10.7% (95% CI 2.8–18.7%, *p* = 0.0106: Table 2, Figure 2). Whereas ScO_2_ was reduced from baseline in the phenylephrine group (*p* = 0.005), this was not the case with the administration of ephedrine (*p* = 0.4657).

**Table 2:** **Change in outcomes from baseline on average during 60 min trial period, in patients randomized to either ephedrine or phenylephrine**.

	**(***n*** = **12**) Ephedrine**	**(***n*** = **12**) Phenylephrine**	**Difference (95% CI)**	***p*-Value**
Frontal lobe oxygenation (%)	2.1 ± 2.8	−8.6 ± 2.8	10.7 (2.8–18.7)	0.0106
Systolic pressure (mmHg)	−19 ± 5	−16 ± 5	−3 (−17 to 11)	0.6329
Diastolic pressure (mmHg)	−17 ± 3	−14 ± 2	−3 (−10 to 3)	0.3093
MAP (mmHg)	−20 ± 3	−14 ± 3	−6 (−15 to 3)	0.1904
Heart rate (bpm)	3 ± 3	−11 ± 3	14 (5–23)	0.0053
Stroke volume (ml)	4.4 ± 5.0	9.7 ± 4.8	−5.3 (−18.9 to 8.3)	0.4299
Cardiac output (L/min)	0.85 ± 0.39	−0.12 ± 0.38	0.97 (−0.08 to 2.02)	0.0683
TPR (dyn s cm^−5^)	−232 ± 42	−129 ± 41	−103 (214 to 8)	0.0684
Fetal heart rate (bpm)	19 ± 3	18 ± 3	1 (−6 to 9)	0.7441

Presented are the ephedrine and phenylephrine group with values in means ± SE (standard error) and differences in mean changes (95% CI). Values for frontal lobe oxygenation (ScO_*2*_) are changes in percent.

### Cardiovascular effects

**Figure 3:**
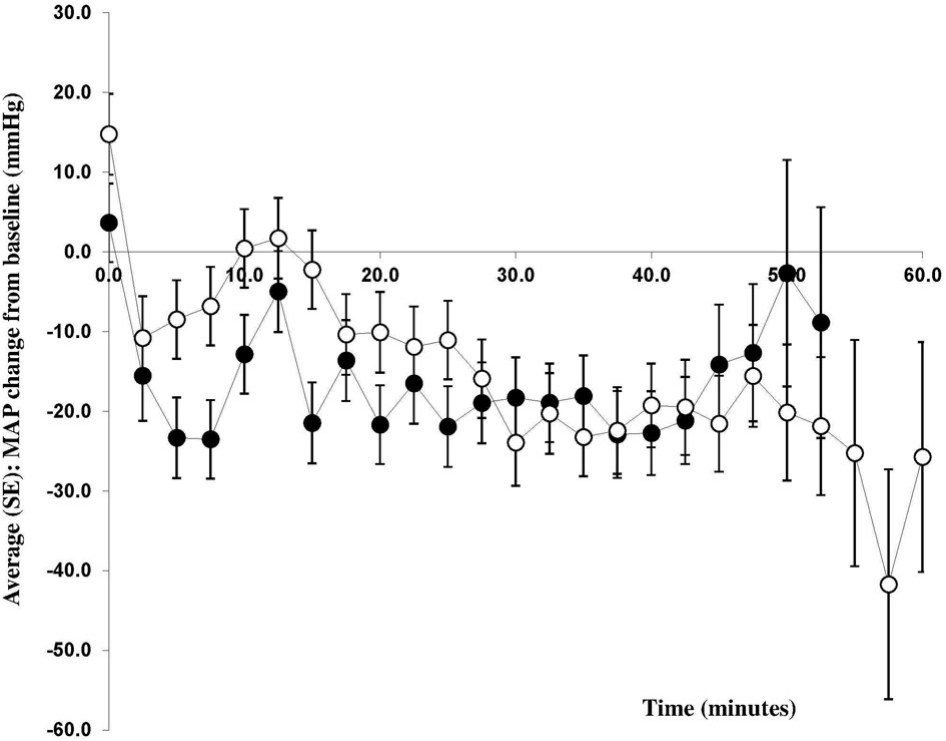
**Change in mean arterial pressure from baseline**. Changes in mean arterial blood pressure (MAP) from baseline (mmHg) during caesarean section with spinal anesthesia. Patients received either phenylephrine (*n* = 12; open circles) or ephedrine (*n* = 12; black circles).

Phenylephrine (−14 ± 3 mmHg) and ephedrine (−20 ± 3 mmHg) were equally effective in maintaining MAP (Figure 3) with a group mean difference of 6 mmHg (95% CI −15 to 3, *p* = 0.1904). Similar results were obtained for DBP and SBP (Table 2). The maternal HR was maintained with ephedrine (+3 ± 3 bpm) but decreased with phenylephrine (−11 ± 3 bpm); difference 14 (95% CI 5–23, *p* = 0.0053) bpm. SV appeared to increase both with phenylephrine (9.7 ± 4.8 ml) and with ephedrine (4.4 ± 5.0 ml), but this difference was not significant: −5.3 ml (95% CI −18.9 to 8.3, *p* = 0.4299). In the ephedrine group CO increased by 0.9 ± 0.4 l/min, while in the phenylephrine group CO was stable (−0.1 ± 0.4 l/min); 95% CI −0.08 to 2.02, *p* = 0.0683). There was a tendency for TPR to be lower in the ephedrine group compared with the phenylephrine group (−232 ± 42 vs. −129 ± 41) dyn s cm^−5^ (*p* = 0.0684).

### Fetal variables

**Figure 4:**
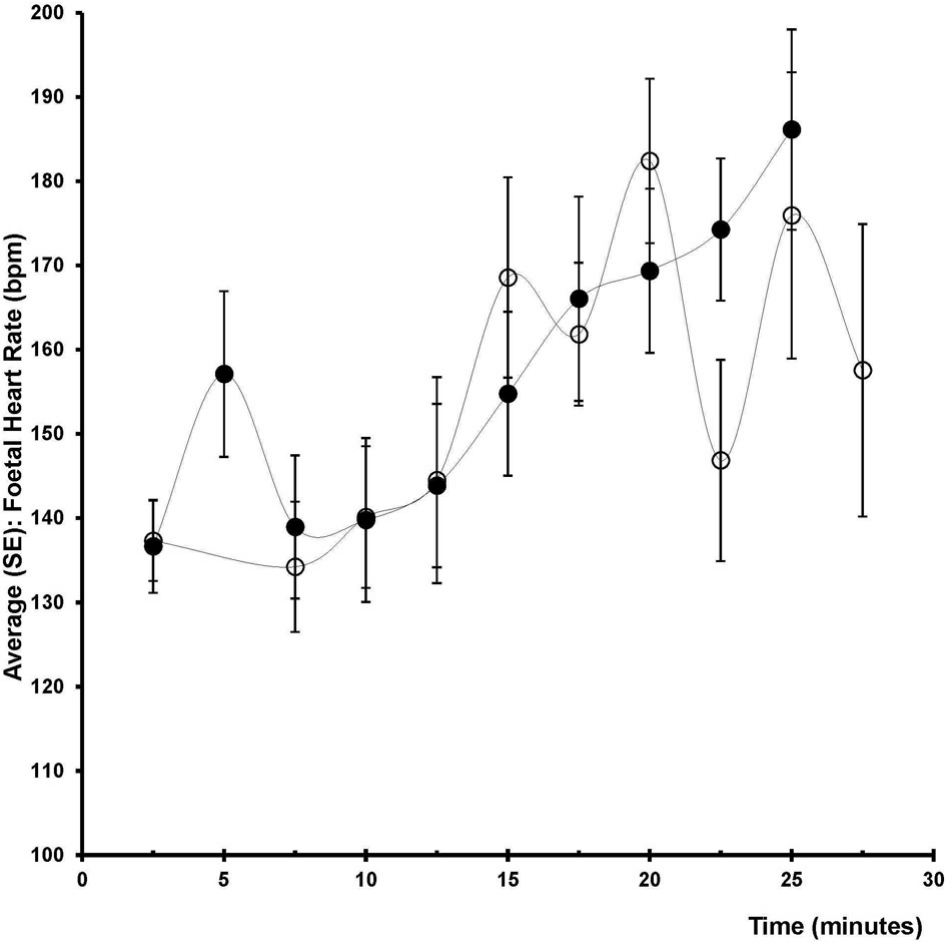
**Foetal heart rate**. Change in fetal heart rate (bpm) after induction (2.5–5 min), before surgery (7.5–17.5 min), after delivery (12.5–27.5 min) in two groups of 12 patients undergoing spinal anesthesia for caesarean section and receiving either phenylephrine (open circles) or ephedrine (black circles).

The difference in fetal heart rate between the ephedrine and phenylephrine group was 1 bpm (95% CI −6 to 9, *p* = 0.7441); for ephedrine +19 ± 3 vs. +18 ± 3 bpm for phenylephrine (Table 2, Figure 4). When compared to baseline both ephedrine (*p* < 0.005) and phenylephrine (*p* < 0.005) increased fetal heart rate. All Apgar scores were = 8 one minute after delivery and increased to 10 after 5 min in both groups. There was a difference (*p* = 0.0223) in regard to the umbilical venous base excess: −1.0 (95% CI −0.86 to −0.14) mM and arterial umbilical lactate was higher in the ephedrine than in the phenylephrine group: 0.50 (95% CI 0.09–0.91) mM, *p* = 0.017, while no differences were observed in any of the other umbilical cord blood variables assessed (Table 3).

**Table 3:** **Umbilical cord blood analysis**.

**Variable**	**Ephedrine**	**Phenylephrine**	**Difference between groups (95% CI)**	***p*-Value**
**UMBILICAL ARTERIAL**				
pH	7.29 ± 0.08	7.31 ± 0.05	−0.02 (−0.07 to 0.04)	0.4699
Pco2; kPa	7.36 ± 1.63	6.98 ± 0.77	0.38 (−0.72 to 1.48)	0.4793
Base excess (mM)**	−0.3 ± 2.8	0.6 ± 1.6	0.9 (−3.0 to 1.1)	0.3414
Lactate (mM)	2.4 [2.2 to 3.4]	1.9 [1.5–2.2]	0.50 (0,09–0.91)^*^	0.0170
**UMBILICAL VENOUS**				
pH	7.37 ± 0.06	7.37 ± 0.04	0.00 (−0.04 to 0.04)	0.9350
Pco2; kPa	5.58 ± 1.00	5.95 ± 0.60	−0.37 (−1.10 to 0.37)	0.3083
Base excess (mM)	−1.0 [−2.6 to −0.5]	0.0 [−0.1 to 0.3]	−1.0 (−0.86 to −0.14)^*^	0.0223
Lactate (mM)	2.1 [1.4–2.6]	1.9 [1.5–2.2]	0.20 (−0,09 to 0.49)^*^	0.1738

Values are mean ± SD and differences in mean changes (95% CI). Exceptions are arterial and venous blood lactate, venous base excess which is shown as median with interquartile range (IQR) in square brackets.

* Median difference.

### Operative results

The time between intra-thecal injection and start of surgery [difference 0.0 (95% CI −2.8 to 2.8) min, *p* = 0.9976)] and between intra-thecal injection and delivery [difference 0.2 (95% CI 0.2 to 3.2) min, *p* = 0.9106)] were similar for the ephedrine and the phenylephrine groups (Table 4). The amount of i.v. saline was also equal between the two groups: 63 ml (95% CI −259 to 134, *p* = 0.0515) and there was no difference in the blood loss (100 ml (95% CI of difference −275 to 475), *p* = 0.601).

**Table 4:** **Surgical times and intravenous fluid**.

**Variable**	**Ephedrine**	**Phenylephrine**	**Difference between groups (95% CI)**	***p*-value**
Induction to incision (min)	13.3 ± 3.3	13.3 ± 3.3	0.0 (−2.8 to 2.8)	0.9976
Induction to delivery (min)	17.5 ± 3.2	17.3 ± 3.9	0.2 (−2.8 to 3.2)	0.9106
Total intravenous fluid (ml)	904 ± 254	967 ± 206	−63 (−259 to 134)	0.5153

Values are mean ± SD and differences in mean changes (95% CI).

### Vasopressor dose

The ephedrine dose per minute was 1.7 mg (1.3–2) range 0.7–5.9 mg and of phenylephrine 1.38 mg (0.91–1.55) range 0.57–1.82 mg. No patient was treated with both phenylephrine and ephedrine.

Further monitoring (not reported) included pulse oximetry, ECG, non-invasive blood pressure on the right arm after initiation of saline administration and continued until the patient was transferred to the postoperative observation unit (no abnormal values were noted).

## Discussion

The present study confirmed that phenylephrine and ephedrine are equally effective in maintaining MAP in patients undergoing caesarean section with spinal anesthesia. The new finding is that in patients undergoing spinal anesthesia, the use of the ephedrine, capable of activating both α and β adrenergic receptors, preserves the near infrared-determined frontal lobe oxygenation (ScO_2_) while ScO_2_ was reduced in patients allocated to administration of phenylephrine, a selective α-adrenergic receptor agonist. We considered that women going through caesarean section during spinal anesthesia might be hypovolemic because of compromised venous return and that phenylephrine therefore could support cardiac output and in turn cerebral oxygenation as expressed by ScO_2_. In contrast to our hypothesis, however, phenylephrine did not increase CO and maybe therefore reduced ScO_2_. In other word, with the established routine for maintaining the circulation during caesarean section during spinal anesthesia, CO in response to administration of phenylephrine indicated that the patients were maintained “normovolemic.” Furthermore, only ephedrine maintained HR and the two drugs elicited an equal almost 20 bpm increase in fetal heart rate.

Spinal anesthesia influences MAP because of sympathetic blockade and during caesarean section inferior caval compression may reduce venous return to the heart and thus CO (Cyna et al., 2006) During caesarean section hypotension is considered when maternal SBP decreases by 20–30% or decreases to less than 90–100 mmHg (Saravanan et al., 2006; Ngan Kee et al., 2008a) although we acknowledge that the lower limit of cerebral autoregulation has been challenged (Lucas et al., 2010; Tan, 2012). Thus, MAP did not fall to what is considered to be the lower limit of cerebral autoregulation (Paulson et al., 1990) and the patients were not expected to be exposed to cerebral hypoperfusion and a reduction in ScO_2_. However, administration of phenylephrine led to a ~9% reduction in ScO_2_ (Figure 2, Table 2). A decrease in MAP to below ~80 mmHg, as provoked for example during head-up tilt, reduces cerebral blood flow and ScO_2_ because of a reduced central blood volume and CO (Madsen et al., 1998) However, whether or not a balance exists between cerebral oxygen supply and demand during anesthesia-induced hypotension remains unknown (Meng et al., 2013).

A 10–15% reduction in ScO_2_ and a 50% reduction in middle cerebral artery mean flow velocity are associated with pre-syncopal symptoms (Kurihara et al., 2007) The 9% reduction in ScO_2_ in response to administration of phenylephrine during spinal anesthesia for elective caesarean section approaches that level and could thus represent clinically important cerebral hypoperfusion (Hunt et al., 2006; Suzuki et al., 2008; Nissen et al., 2009b). On the other hand, the patients were awake and able to report hypotension-associated symptoms (nausea, vomiting or dizziness), but no complaints were expressed regardless of the NIRS value. It is possible that the 9% reduction in ScO_2_ following the administration of phenylephrine reflects contamination of the NIRS signal from extracranial tissue (Davie and Grocott, 2012).

There was no difference in fetal HR among groups of patients and neonatal outcome was similar, although there were small but probably clinically unimportant differences in regard to umbilical arterial lactate and venous base excess. Ephedrine could increase fetal HR due to β-adrenergic stimulation, when or if it crosses placenta inducing fetal acidaemia (LaPorta et al., 1995) The impact of ephedrine on arterial lactate and venous base excess could be related to an effect on uteroplacental or feto-placental circulation (McGrath et al., 1994; LaPorta et al., 1995).

We recognize that this evaluation is not a blinded randomized controlled trial and further that it is a limitation to the study that both physicians and nurses expected that ephedrine would increase maternal heart rate and therefore potentially influenced by that expectation.We estimated blood pressure 2.5 min after initiation of anesthesia and with the use of vasopressor this considered as a long time and could represent a limitation of the study.

There were some difficulties in using Nexfin to estimate cardiovascular variables during caesarean section when the patients moved due to anxiety. Also, Modelflow may not be provide an accurate CO (Remmen et al., 2002) and calibration by thermodilution (Jansen et al., 2001) or by the Fick method (van Lieshout et al., 2001) may be in need. However, for tracking changes in CO the Nexfin has been successfully validated against a thermodilution estimate during a deliberate reduction in central blood volume induced by standing up in healthy subjects (Harms et al., 1999) during cardiac surgery (Jansen et al., 2001) in intensive care medicine (Jellema et al., 1999) and during liver transplantation (Nissen et al., 2009c).

Phenylephrine preserved MAP and reduced HR and maintained CO with a slight increase in SV that was 5 ml higher than the increase in the subjects receiving ephedrine. Phenylephrine may increase cardiac afterload to an extent that SV and CO decrease. On the other hand, an increase in vascular tone can increase venous return and thereby cardiac preload, SV and CO during hypovolemia as demonstrated in anesthetized pigs (Cannesson et al., 2012) Meng et al. found an increase in SV and CO after administration of phenylephrine using pulsewave analysis by Vigileo-FloTrac in non-pregnant patients, but the increases were not confirmed with the use of a trans-esophageal Doppler apparatus (Meng et al., 2011b). Also an increase in SV in response to administration of phenylephrine is supported by Doherty et al. who used a CO monitor based on bi-reactance technology (Doherty et al., 2012) and by Dyer et al. using a LiDCOplus monitor (Dyer et al., 2009).

Another limitation of this trial was that we measured ScO_2_ and not cerebral blood flow, but parallel variation between middle cerebral artery mean flow velocity in basal cerebral arteries and ScO_2_ is reported (Ide et al., 1999; Yoshitani et al., 2007; Steiner et al., 2009) Furthermore, determination of internal jugular venous oxygen saturation could validate if the data obtained by NIRS are representative for the whole brain (Kim et al., 2000).

Three different NIRS approaches can be implemented: (1) Continuous wave (CW), (2) Frequency-domain (FD), and (3) time domain technology (TD). An approximate 3–4% decrease in ScO_2_ is reported after treatment with phenylephrine with the use of FD (Meng et al., 2011a), compared with the 9% decrease in this evaluation while others (Nissen et al., 2010; Ogoh et al., 2011) find an approximate decrease by 14% using CW. With commercial CW monitors there is a variation in the ability to estimate ScO_2_ (Davie and Grocott, 2012).

## Conclusion

This study confirmed that infusion of phenylephrine and ephedrine are equally effective for sustaining blood pressure during elective caesarean section with spinal anesthesia. Phenylephrine, however, reduced ScO_2_ and maternal heart rate when compared to ephedrine but the two drugs induced an equal increase in fetal heart rate.

## Author's contribution

Peter Nissen, Niels H. Secher, Henning B. Nielsen, Kim Z. Rokamp, and Visti T. Foss conceived and designed the trial protocol. Visti T. Foss procured the project funding in cooperation with Department of Anaesthesia, Næstved Hospital, Denmark. Visti T. Foss and Kim Z. Rokamp contributed to clinical screening and recruitment of patients. Visti T. Fossand Kim Z. Rokamp handled the cooperation with the Good Clinical Practice (GCP) unit at the University of Copenhagen with VF as primary investigator and Kim Z. Rokamp as sponsor. Visti T. Foss and Robin Christensen did the statistical analyses. Visti T. Foss, Robin Christensen, Niels H. Secher and Kim Z. Rokamp drafted the manuscript, and Peter Nissen and Henning B. Nielsen contributed to the manuscript. All authors read and approved the final manuscript. Visti T. Foss and Kim Z. Rokamp accept full responsibility for this work and act as guarantors for the study.

### Conflict of interest statement

The authors declare that the research was conducted in the absence of any commercial or financial relationships that could be construed as a potential conflict of interest.
